# Brain Network Organization Correlates with Autistic Features in Preschoolers with Autism Spectrum Disorders and in Their Fathers: Preliminary Data from a DWI Analysis

**DOI:** 10.3390/jcm8040487

**Published:** 2019-04-10

**Authors:** Lucia Billeci, Sara Calderoni, Eugenia Conti, Alessia Lagomarsini, Antonio Narzisi, Camilla Gesi, Claudia Carmassi, Liliana Dell’Osso, Giovanni Cioni, Filippo Muratori, Andrea Guzzetta

**Affiliations:** 1 Institute of Clinical Physiology, National Research Council of Italy, 56124 Pisa, Italy; lucia.billeci@ifc.cnr.it; 2 IRCCS Stella Maris Foundation, 56128 Pisa, Italy; econti@fsm.unipi.it (E.C.); anarzisi@gmail.com (A.N.); gcioni@fsm.unipi.it (G.C.); fmuratori@fsm.unipi.it (F.M.); aguzzetta@fsm.unipi.it (A.G.); 3 Department of Clinical and Experimental Medicine, University of Pisa, 56126 Pisa, Italy; alessia.lagomarsini@gmail.com (A.L.); camillagesi@hotmail.com (C.G.); ccarmassi@gmail.com (C.C.); liliana.dellosso@med.unipi.it (L.D.)

**Keywords:** autism spectrum disorders, preschoolers, diffusion tensor imaging, brain connectivity, connectome, fathers, broader autism phenotype

## Abstract

Autism Spectrum Disorders (ASD) is a group of neurodevelopmental disorders that is characterized by an altered brain connectivity organization. Autistic traits below the clinical threshold (i.e., the broad autism phenotype; BAP) are frequent among first-degree relatives of subjects with ASD; however, little is known regarding whether subthreshold behavioral manifestations of ASD mirror also at the neuroanatomical level in parents of ASD probands. To this aim, we applied advanced diffusion network analysis to MRI of 16 dyads consisting of a child with ASD and his father in order to investigate: (I) the correlation between structural network organization and autistic features in preschoolers with ASD (all males; age range 1.5–5.2 years); (II) the correlation between structural network organization and BAP features in the fathers of individuals with ASD (fath-ASD). Local network measures significantly correlated with autism severity in ASD children and with BAP traits in fath-ASD, while no significant association emerged when considering the global measures of brain connectivity. Notably, an overlap of some brain regions that are crucial for social functioning (cingulum, superior temporal gyrus, inferior temporal gyrus, middle frontal gyrus, frontal pole, and amygdala) in patients with ASD and fath-ASD was detected, suggesting an intergenerational transmission of these neural substrates. Overall, the results of this study may help in elucidating the neurostructural endophenotype of ASD, paving the way for bridging connections between underlying genetic and ASD symptomatology.

## 1. Introduction

Autism Spectrum Disorders (ASD) is a heterogeneous group of neurodevelopmental conditions, which is characterized by impairments in socio-communication and the presence of restricted/repetitive behaviors [1], with a consistent prevalence rate in different countries of about 1% of children [2,3,4]. Although the exact etiopathogenesis of ASD remains elusive in the majority of cases, a combination of genetic predisposition, environmental influence, and the interaction between the two has been repeatedly suggested [5,6]. Regarding genetic influences, the analysis of single nucleotide variants (SNVs) on the whole genome (genome-wide association studies, GWAS) [7,8,9], of copy number variants (CNVs) [10], and the sequencing of the whole exome in subjects with ASD [11,12] detected a large set of rare variants, highly penetrating, affecting hundreds of genes. Crucially, the several hundred genes that are involved converge on the functioning of a smaller number of key pathways, such as development and activity of synapses (molecules of neuronal adhesion, synaptic transmission, activity-dependent protein synthesis), regulation of transcription, and remodeling of the chromatin [13,14]. Genetic anomalies are implicated in a considerable proportion of ASD cases (at least 10–20%). For the remaining ~85% cases, in which specific genetic variations are not yet detectable, the sources of ASD heritability remain to be clarified.

Consistent with a genetic predisposition in ASD, several studies have reported that, in about 25% of families of persons with ASD, other family members display various ASD manifestations, ranging from full–blown autistic disorder, autistic symptoms, or autistic traits [15,16,17], suggesting that ASD and autistic traits might share common underlying genetic susceptibility factors [18]. More specifically, literature consistently suggests that parents of individuals with ASD (pASD) are more likely than the general population to express personality traits similar, although less severe, to those of ASD individuals, i.e., the so-called broad autism phenotype (BAP) [19]. These sub-threshold characteristics include peculiar social, communication, and cognitive processes, strong persistent interests, and rigid and aloof personality traits [20,21], and they are higher in fathers when compared to mothers of individuals with ASD (for a systematic review, see [22]). Still little is known, however, on whether genetic and behavioural commonalities between pASD and their sons with ASD also mirror at the brain level. This issue is of relevance, since neuroimaging could reveal ‘intermediate phenotypes’ or ‘endophenotypes’ that are more closely associated with specific genes than the clinical phenotype, and they can therefore support the discovering of new disease genes or the characterization of genetic subtypes of the disease [23].

To this aim, some investigations have examined the neurostructural and the neurofunctional underpinnings in pASD. In a pioneering single case report, Volkmar and colleagues [24] reported similar MRI abnormalities in the dorsolateral frontal region in a father and his 15-year-old son with Asperger syndrome. More recently, studies regarding this topic were summarized in a review [25]. The selected studies used structural magnetic resonance imaging (sMRI), magnetic resonance spectroscopy (MRS), functional magnetic resonance imaging (fMRI), electroencephalography (EEG), or magnetoencephalography (MEG) to explore neurobiological substrates of pASD. The results indicated that pASD are generally different from healthy controls at a structural [26,27] and functional level [28,29]. In addition, a positive correlation between neuroanatomical characteristics and BAP traits is emerging [30,31]. Crucially, some of the observed atypicalities involve the same brain regions (e.g., fusiform gyrus) as the ASD probands, suggesting a potential genetic influence [30]. Moreover, gender was found to influence the neurostructural and neurofunctional results: in particular, the neuroimaging study of Baron-Cohen et al. [32] supported the “Extreme Male Brain Theory” of ASD [33], with mothers of individuals of ASD expressing even less fMRI activation than male controls in empathic tasks.

To the best of our knowledge, no study has used diffusion-weighted imaging (DWI) to explore structural connectivity in pASD and its relationship with behavioral measures. DWI allows for indirect inferences on anatomical connectivity based on differential water diffusion [34], and it is thought to be particularly suited to assess the neuroanatomical underpinnings of ASD, which has been increasingly considered a brain connectivity disorder [35,36,37]. This assumption stems from the several DWI-based studies that were performed in the last two decades to investigate the white matter tracts of individuals with ASD, which revealed a complex pattern of abnormalities in brain connectivity when compared to matched controls [38]. Specifically, atypical connectivity in a distributed network of brain regions specialized in understanding the social behaviours of others—the so-called ‘social brain’—is thought to be involved in social impairment, which is a cardinal feature of the autistic spectrum. This circuit comprises a set of areas that are implicated in processing social stimuli, i.e., the orbitofrontal and medial prefrontal cortices, the superior temporal cortex, the temporal poles, the amygdala, the precuneus, the temporo-parietal junction, the anterior cingulate cortex (ACC), and the insula [39]. Atypical connectivity patterns in ASD are not limited to the social brain, but it also includes thalamo-frontal [40], fronto-striatal [41], cerebellar [42], and motor-sensory homunculus [43] connections, as well as corpus callosum [44]. Further, a developmental trend in the disruption of brain structural connectivity was detected. In fact, infants and toddlers with ASD are characterized by a predominance of over-connectivity pattern when compared with age-matched controls [45,46,47,48], while adolescents and adults predominantly show an overall under-connectivity, as to matched peers [49,50]. However, recent studies revealed a picture that is more complex, in which over- and under-connectivity are network-dependent and may coexist in the brain of subjects with ASD, independently from their age [51].

In the current study, we performed brain structural connectivity in ASD children and in their fathers by applying the HARDI (High Angular Resolution Diffusion Imaging) protocol. This method is able to reduce the known limits of the diffusion-weighted MRI, allowing for a better identification of crossings and branching fibers, which are highly prevalent in brain white matter [52]. In addition, the adoption of a mathematical approach based on graph theory allowed us to examine the brain as a network of interconnected processing units, rather than exploring individual anatomical connections [53]. Graph analysis approaches use measures of the length and strength of connections between all pairs of brain regions to evaluate the efficiency of information transfer within the network, assessing how brain abnormalities impact communication, both at the global and local level. The primary aim of this study was to more specifically explore brain-behavior correlations in the two groups: (I) to correlate autistic features in terms of Autism Diagnostic Observation Schedule Second Edition (ADOS-2) [54] scores with structural network organization in ASD preschoolers; (II) to correlate BAP features in terms of Autism-Spectrum Quotient (AQ) [55] with structural network organization in fathers of ASD preschoolers (fath-ASD). The secondary aim was to test the hypothesis that regions that were identified in fath-ASD may overlap those identified in their probands. We restricted our investigation to male subjects with ASD and their fathers, as fathers have higher rates of BAP than mothers [22], and gender can impact on neuroanatomical findings, both in typical [56] and ASD subjects [57].

## 2. Experimental Section

### 2.1. Participants

Sixteen ASD-child/father dyads (all Caucasian) were recruited at IRCCS Stella Maris Foundation (Pisa, IT). The study protocol was approved by the Pediatric Ethic Committee of the Tuscany Region and was performed in accordance with the Declaration of Helsinki. A document with all the necessary information about the study protocol as well as a written informed consent form to participate in the study were given and signed by the parents.

The inclusion criteria for children were an age-range between 18 and 72 months and male gender. Fathers were only included if they were 18 years or older. Exclusion criteria for both fathers and their children were: (I) brain anomalies that were detected on MRI; (II) neurological syndromes or focal neurological signs; (III) history of birth asphyxia, extreme premature birth (≤28 gestational weeks) or perinatal insult; (IV) epilepsy; (V) significant sensory impairment (e.g., blindness, deafness); (VI) use of any psychotropic medication; and, (VII) contraindication for MRI. Additional exclusion criteria for fathers were a poor comprehension of Italian language, which could have biased the clinical evaluation, and insufficient cooperation for MRI scans. Children with ASD also performed the recommended laboratory tests to rule-out medical causes of ASD, including audiometry, thyroid hormone disorders, DNA analysis of FRA-X and screening tests for inborn errors of metabolism (plasma and urine aminoacid analysis, urine organic acid measurement, urine mucopolysaccarides quantitation, plasma and urine creatine, and guanidinoacetate analysis).

Table 1 reports on the demographic and clinical characteristic of the participants.

### 2.2. Clinical Assessment

All of the children received a clinical diagnosis of ASD according to DSM-5 criteria [1] that was confirmed using algorithm cutoffs on the ADOS-2 [54], as administered by an evaluator (A.N.) who has obtained research reliability certification. Children with ASD perform structural MRI as part of the clinical assessment protocol with the aim of excluding brain alterations. In the case of absence of anomalies detected on MRI, the father of the child was asked to participate in the study and was evaluated with the same MRI protocol.

ADOS calibrated severity score (ADOS-CSS) was used as a clinical measure of ASD severity in children. Separate severity metrics for the Social Affect (SA) and Restricted, Repetitive Behavior (RRB) domains were also considered, which could provide a better picture of ASD dimension [58]. In addition, all children with ASD were assessed for non-verbal development quotient through the performance subscale of the Griffiths Mental Developmental Scales (GMDS).

The fathers enrolled in the study were evaluated through a semi-structured clinical interview that aimed at making the major psychiatric diagnoses (SCID-I, [59]) and through a series of self-administered questionnaires to evaluate post-traumatic stress disorder (Trauma and Loss Spectrum-self report, TALS-SR lifetime version) [60,61] and mood disorders (Mood Spectrum-self report, MOODS-SR—lifetime version) [62].

BAP traits evaluation was based on the Autism-Spectrum Quotient (AQ) [55], a self-report questionnaire that evaluates the following five different areas: “social skills”, “attention switching”, “attention to detail”, “communication”, and “imagination”.

### 2.3. Image Acquisition

Structural and diffusion tensor MRI were acquired on a 1.5 T MR system (Signa Horizon LX, GE Medical System). Children were scanned under bland sedation. They received inhalational anesthesia with an odorless oxygen and nitrous mixture for induction and sevoflurane for maintenance. No side effects were reported. The fathers were scanned while they were awake, after being recommended to stay as still as possible during the scan acquisition. The MRI protocol included an axial MRI three-dimensional (3D) brain volume (BRAVO) T1-weighted (acquisition matrix = 256 × 256, TR/TE = 12,332/5.16 ms, voxel dimension = 0.5 × 0.5 × 2 mm^3^, field of view = 256 mm) and an HARDI scan that was acquired along 30 uniformly distributed diffusion encoding directions (b = 1000 s/mm^2^), along with one b = 0 image (acquisition matrix: 80 × 80, voxel dimension = 3 × 3 × 3 mm^3^, TR/TE = 10,000/92 ms, field of view = 240 mm). Notably, in a HARDI approach, the diffusion-weighted images are acquired using a large number of non-collinear encoding directions (ideally 60 or above); however, crossing fibers can also be resolved using 30-direction diffusion data, albeit less accurately [63].

### 2.4. Structural Data Analysis

Structural MRI segmentation was performed in native space using the FreeSurfer software package [64]. Although Freesurfer analysis is not formally recommended for use in children under four years of age due to insufficient gray–white matter contrast, it has been previously used in investigations in infants and toddlers as young as 12 months [46,65,66,67]. Images were visually inspected at each stage in the Freesurfer processing pipeline and, if needed, manually edited and corrected to avoid errors in the segmentation procedure. This included inspecting data for poor skull-stripping, the additional use of “*gcut*” (http://freesurfer.net/fswiki/FsTutorial/SkullStrip_Fix_freeview) and, in extreme cases, the manual removal of remaining dura, eye, and other non-brain signal. Using this controlled procedure, we also obtained good results for younger children.

FreeSurfer provides parcellation of anatomical regions of the cortex (34 for each hemisphere) based on the Desikan atlas [68] and subcortical regions [69], eight for each hemisphere (nucleus accumbens, amygdala, caudate, hippocampus, pallidum, putamen, and thalamus and cerebellum) were included. Thus, the final parcellation, including both hemispheres, consisted of 84 cortical and subcortical regions in total (Table 2). Cortical regions are defined according to the Desikan atlas [68] and subcortical regions, according to Fischl et al. [69]. The Freesurfer procedure has been previously validated in a control group of typically developing children [70].

**Figure 1:**
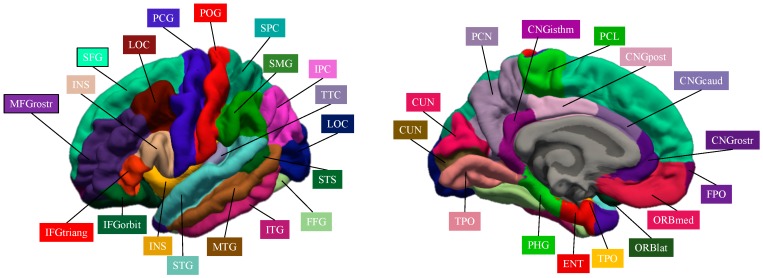
Cortical brain regions obtained by Freesurer parcellation.

Figure 1 represents the cortical brain regions that were obtained by Freeserfer parcellation.

### 2.5. Diffusion Data Analysis and Connectome Construction

An extensive pre-processing procedure was applied to HARDI data to correct for head motion, image distortions, and artifacts based on previous work [70]. In brief, first volumes with motion between subvolumes were detected and removed based on a discontinuity index [71]. Subsequently, outlier detection and replacement, correction for distortions (eddy currents and subject motion) was performed used the integrated new tool “*eddy*” implemented in FSL version 5.0.11 [72].

Spatial intensity inhomogeneities were reduced using N3 correction [73]. The bias field was calculated from the image with b = 0, and subsequently applied to all diffusion-weighted images. Using MRtrix3 software (http://www.mrtrix.org) tensor, the fractional anisotropy (FA) and mean diffusivity (MD) maps were successively computed and the fiber orientation distribution (FOD) was estimated using single-tissue constrained spherical deconvolution while applying an algorithm that is a reimplementation of the iterative approach proposed in Tournier et al. [74].

Tractography was performed using an approach that was based on the integration of anatomically-constrained tractography (ACT) [75], which uses anatomical information from high-resolution T1-weighted images to control the evolution and termination of fiber tracking and spherical-deconvolution informed filtering of tractograms (SIFT) [76].

First, registration between structural and diffusion data was performed using the Boundary-Based Registration (BBR) approach [77]. The T1-weighted image that was generated by Freesufer processing was used as it is isotropic (1 × 1 × 1 mm^3^). Subsequently, tissue partial volume maps of brain white matter (WM), cortical grey matter (GM), deep GM, and cerebrospinal fluid (CSF) were prepared for the ACT framework while using FSL tools [78]. For each scan, tractograms of 10 million streamlines were generated through seeding from either WM mask or deep GM. SIFT was finally applied to filter the reconstruction from 10 million to five million streamlines.

Using MRtrix3 software, the connectomes were generated while considering all possible connecting streamlines between each pairs of nodes. Connectomes were generated using streamline count as the weighting factor.

**Figure 2:**
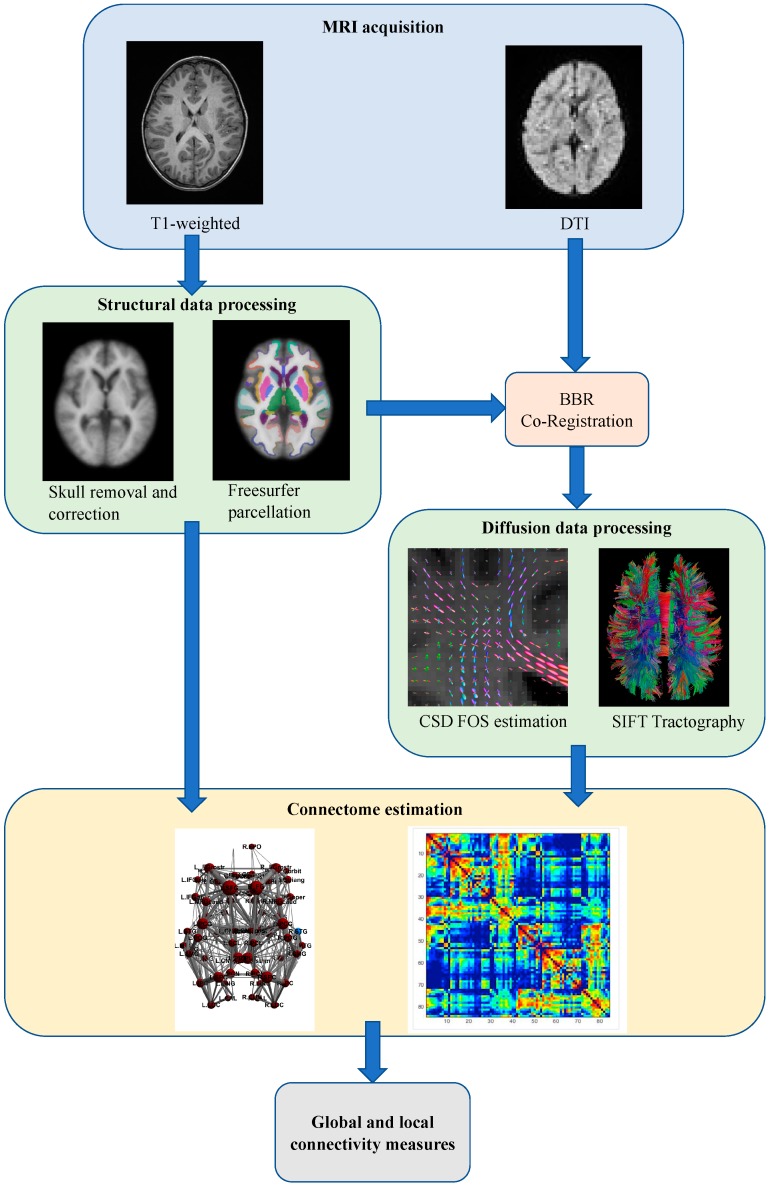
Overall schematic representation of the construction of the structural brain networks. BBR: Boundary-Based Registration; CSD: constrained spherical deconvolution; FOD: fiber orientation distribution; SIFT: spherical-deconvolution informed filtering of tractograms.

Figure 2 schematically represents the overall analysis procedure.

### 2.6. Graph Analysis of Connectomes

The graph theory approach was used to analyze connectome. First, a group threshold of 60% was applied to connectomes in order to eliminate false positive and false negatives [79]. The threshold was separately applied for the group of fathers and the group of children.

The Brain Connectivity Toolbox [80], as well as homemade Matlab scripts, were then used to perform the analyses. Global measures (network-level) and local measures (node-level) were calculated for each connectome. The global measures computed were the global efficiency (EG) [81], the transitivity (T) [82], and the characteristic path length [83]. In addition, Small-World Propensity (SWP), a measure to evaluate small-world characteristics in weighted neural networks [84], was calculated. The local measures used in this study were local efficiency (LE) [81], clustering coefficient (CC) [83], and betweenness centrality (BC) [85]. Table 3 reports the graph theoretical measures extracted in this study. For a more detailed description, please see Bullmore and Sporns [53] and Rubinov and Sporns [80].

### 2.7. Statistical Analysis

Statistical analyses of the data were performed using SPSS software for Mac Version 20.0 (SPSS Inc, Chicago, IL, USA). Multiple univariate general linear model (GLM) based variance analysis was performed for each brain region, in which the network measures were dependent variables and the clinical variables were independent variables, to identify the clinical correlates of the network characteristics. Age was regressed out as covariate, as it has been previously demonstrated that it can influence the diffusion-weighted MRI parameters [86].

In the group of children, the ADOS-CSS, the SA and the RRB domains, and the GMDS were evaluated, while in the group of fathers the AQ total score as well as its subdomains were considered as the clinical variables. The effect sizes were estimated by partial eta squared (η^2^; values between 0.01 and 0.06 are they generally considered to be a small effect, between 0.06 and 0.14 a medium effect, and those above 0.14 are regarded as a large effect) [87]. A multiple comparison correction using the Benjamini–Hochberg procedure for false discovery rate (FDR) control [88], with a level set at 0.05 was applied, resulting in a significance of *p* < 0.0083 (0.05/6 clinical variables). However, due to the explorative nature of the study, we also reported significance values with *p* < 0.05.

## 3. Results

### 3.1. Correlation between ASD Symptoms and Brain Network Measures in ASD Probands

#### 3.1.1. Global Measures

Using multiple linear regression analyses, no significant correlations emerged between clinical features and global measures in ASD probands. A significant positive correlation between age and SWP was found (F = 9.78, *p* = 0.02, η^2^ = 0.620).

#### 3.1.2. Nodal Measure

Significant correlations were found for the nodal measures and the clinical measures. We report here only those that survived FDR correction and those obtained for the same brain areas in fathers and children. Supplementary Data S1 reports all of the significant correlations.

EL index. ADOS-CSS was significantly positively correlated with EL of right PCL, left CNGpost, and right STG. SA was also positively correlated with the left CAU. Age also showed a significant negative correlation with EL of left CAU and of right STG.

CC index. For CC, significant correlations in most of the same areas were found. ADOS-CSS was positively correlated with CC of CNGpost. Regarding ADOS subdomains, SA was positively correlated with left CAU, right FPO, and right ITG. GMDS was positively correlated with right ITG.

BC index. ADOS-CSS was significantly positively correlated with the BC of right PCG. SA was significantly positively correlated with the BC of right MFGcaud, left CNGpost, and significantly negatively correlated with the BC of the left AMY. RRB was negatively correlated with the BC of the the left CNGpost. Age showed a significant negative correlation with left AMY.

Table 4, left column, summarizes the significant correlations between ASD symptoms and brain network measures in ASD probands.

### 3.2. Correlation between BAP Traits and Brain Network Measures in Fathers of ASD Probands

#### 3.2.1. Global Measures

No significant correlations were found between global measures and autistic traits in fath-ASD

#### 3.2.2. Nodal Measures

Significant correlations were found for the nodal measures and the clinical measures. No significant correlation between the nodal measures and total AQ were found. Conversely, the following significant correlations were observed with the AQ subdomains.

As for children, we only report those that survived FDR correction and those that were obtained for the same brain areas in fathers and children, while Supplementary Data S1 reports all of the significant correlations.

EL index. The “attention switching” area of the AQ was negatively correlated with the EL of left CNGisthm and right STG.

CC index. Additionally, for the CC, the “attention switching” area of the AQ was negatively correlated with several brain regions: right SFG, right MFGrostr, left FPO, left LOC, left CNGisthm, right SMG, and right ITG.

BC index. Regarding the BC a significant positive correlation between the “social skills” area of the AQ and the BC of the right THA, right MFGcaud, and right MTG was detected. The “imagination” area of AQ was also significantly positively correlated with the BC of the left IFGoperc. Moreover, “imagination” was negatively correlated with BC of left LOC, left CNGisthm, and right AMY. Age significantly correlated with the BC of left POG.

Table 4, the right column, summarizes the significant correlations between BAP traits and brain network measures in fath-ASD.

### 3.3. Brain Areas Shared in Fathers and in Their ASD Probands

When considering the correlations that were reported in the above paragraphs, it emerges that fathers and their ASD probands shared some brain areas of significance i.e., areas that in both groups are correlated with BAP/clinical measures. These brain areas are highlighted in bold in Table 4 and summarized in Table 5.

In particular, EL of right STG and left CNG was correlated to BAP/clinical measures in both fathers and their ASD probands. CC of right ITG and left CNG and FPO (right in ASD probands and left in their fathers) also expressed significant correlations in both groups.

Finally, the BC of left LOC, right MFGcaud, left CNG, and AMY (left in ASD probands and right in their fathers) significantly correlated with BAP/clinical measures in the two groups.

**Figure 3:**
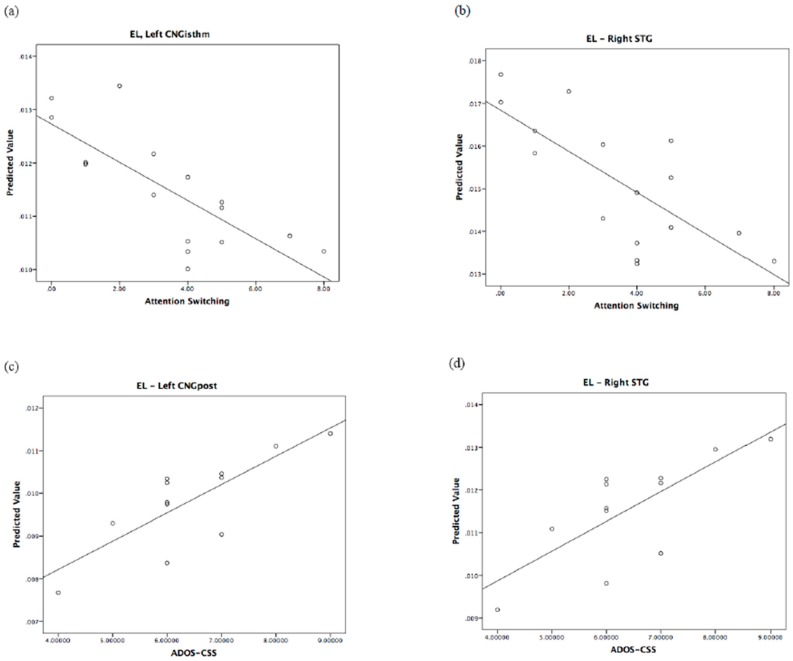
Regional local efficiency correlates of autistic traits in fathers and of autism severity in children. Top: predicted values vs. AQ domains in fathers. Bottom: predicted values vs. ADOS in children. EL: local efficiency.

**Figure 4:**
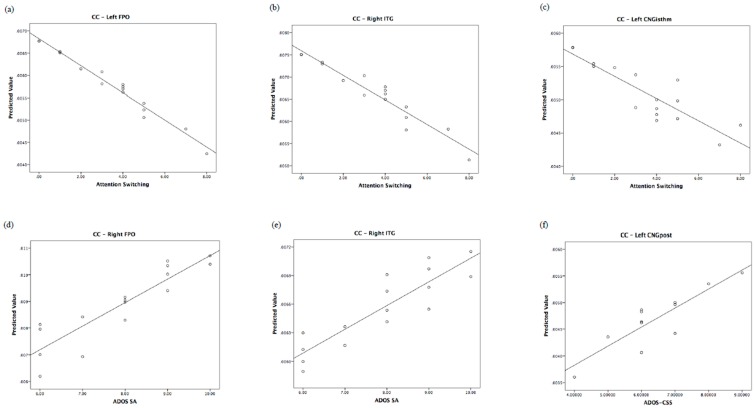
Regional clustering coefficient correlates of autistic traits in fathers and of autism severity in children. Top: predicted values vs. AQ domains in fathers. Bottom: predicted values vs. ADOS in children. CC: clustering coefficient.

**Figure 5:**
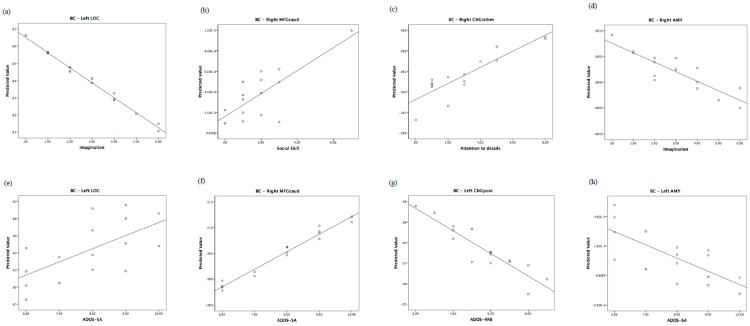
Regional betweenness centrality correlates of autistic traits in fathers and of autism severity in children. Top: predicted values vs. AQ domains in fathers. Bottom: predicted values vs. ADOS in children. BC: betweenness centrality.

The relationship of the predicted and observed values of graph measurements for those brain regions shared by children and their fathers is represented in Figure 3, Figure 4 and Figure 5. In order to understand whether all of the couples showed the same degree of correlation, we performed an analysis of residuals of GLM, which is reported in Supplementary Data S2.

To better visualize the anatomical regions that were significantly associated with autistic traits in fathers and in their children, we plotted connectivity graphs which are reported in Supplementary Data S3.

## 4. Discussion

The primary aim of this investigation was to explore the brain-behavior correlations in ASD preschoolers and in their fathers (fath-ASD) by using HARDI diffusion techniques. To the best of our knowledge, this is the first study applying an advanced diffusion tractography approach to explore this possible association. The results indicated that the local network measures significantly correlated with autism severity in ASD children and with BAP traits in fath-ASD, while no significant association emerged when considering the global measures of brain connectivity. Interestingly, in ASD probands, correlations were mainly positive, while in fath-ASD correlations, they were mostly negative (especially for local efficiency and cluster coefficient).

### 4.1. Correlations between Autistic Severity and White Matter Measures in Children

ASD children mostly displayed a positive association between local efficiency/clustering coefficient and ASD severity (ADOS-CSS and SA), thus suggesting higher connectivity indexes in most impaired subjects. In particular, the brain regions that are involved have been previously associated with ASD impairments, like the caudate [89], known to be implicated both in sensorimotor and cognitive functions [90], or the cingulum [45], associated to empathic cognition, social behaviour, and pain perception [91]. This result agrees with other studies reporting a positive correlation between the connectivity and severity of the social domain [92,93].

A positive correlation between GMDS and local efficiency as well as the clustering coefficient in children with ASD was also observed. This is consistent with the recent report of a significant positive association between local connectivity and language performance in individuals with ASD [94]. We could speculate that this result is in line with the compensatory theory [95,96], according to which, during the development, neural reorganization of brain networks (e.g., high local connectivity) may occur as a compensatory strategy, and may result in better performances.

Interestingly, we found a correlation in the opposite direction for the RRB symptoms, suggesting the existence of a mix pattern of both over- and under-connectivity that subtended specific autistic domains, which is in agreement with previous investigations [97,98]. Notably, in our study, such an inverse, negative correlation was found between local efficiency or cluster coefficient of hippocampus and inferior parietal cortex. Studies in the animal models of ASD have related hippocampal dysfunction to restrictive and repetitive behaviors (RRB) [99,100], while the inferior parietal cortex has been previously associated with cognitive flexibility [101]. Also, RRB have been correlated with weaker brain connectivity in adolescents with ASD [102], and with stronger brain connectivity in adults with ASD [103]. These results are consistent with our findings, and they suggest that, at a younger age, RRB are associated with both reduced local efficiency and cluster coefficient, and thus with weak local connectivity.

### 4.2. Correlations between BAP Traits and White Matter Measures in Fathers

Following the concept of BAP, some studies have explored whether the same neural alterations that were observed in ASD individuals are present in non-clinical or in ASD first-degree relatives. Several techniques, including fMRI, EEG, and MEG have been utilized to assess the brain correlates of autistic traits in pASD, however MRI tractography was never applied [25]. Few studies have used diffusion-weighted MRI to assess the relationship between autistic traits and white matter microstructure in a non-clinical sample of adults, detecting significant associations. For example, Gibbard et al. [104], in a combined sample of individuals with and without ASD, have reported significant negative correlations between the fractional anisotropy (FA) values in several brain regions and AQ score. Hirose et al. [105] found that autistic traits in healthy adults were significantly negatively correlated with the FA in regions that are related to core features of ASD. Positive brain-behavior associations were also reported. For instance, in the study by Iidaka et al. [106], autistic traits were positively associated with the volume of connectivity between superior temporal gyrus and amygdala, while, in the study by Bradstreet et al. [107], autistic traits were positively correlated with FA values in left inferior longitudinal fasciculus. Notably, both significant and positive associations with autistic traits have been reported in the study by Takeuchi et al. [108], which explored WM structural correlates of empathizing and systemizing in young, typically developing adults.

Only one study used a graph theoretical network approach to investigate the functional connectivity of autistic traits in a population of typically developing individuals [109]. Again, both positive and negative correlations between autistic traits and local measures of functional connectivity were found. In particular, the two questionnaires that were used to assess autistic traits (the Social Responsiveness Scale and the Autistic Spectrum Screening Questionnaire) often showed opposite correlations, possibly capturing different aspects of the ASD endophenotype.

Overall, regions we found to be significantly correlated with BAP traits in fath-ASD mostly overlap with those previously that were reported as correlated to autistic traits in non-clinical samples [105,106,110,111]. Importantly, while most previous studies were only able to identify correlations with the total score of the questionnaire, in the current investigation we found significant correlations with the AQ subdomains. This finding may be explained by the different approach that was used in our study (graph analysis versus more traditional DWI techniques), but also by the fact that fath-ASD generally have more autistic traits than typical adults [17]. Accordingly, a wider network of altered regions than those that were recognized in previous diffusion-weighted MRI studies of typical adults was identified. In agreement with the study by Jakab et al. [109], we did not observe any significant correlation with global efficiency, but we detected significant correlations with local measures in several brain regions, suggesting that the impairment of connectivity could be regionally specific. In particular, most of the correlations that we obtained were negative, suggesting that fath-ASD, such as adults with ASD, present higher expression of autistic features in association with lower connectivity indexes. However, some sporadic opposite trends were also found (in particular for “communication” and “attention to detail” domains) [108,109].

Some of the regions that we identified as implicated in autistic traits (i.e., LOC, INS, AMY, SMG, STG) overlap with those that were identified as atypical in previous structural and functional studies of pASD [25]. For example, Yucel et al. [31] investigated neural substrates of faces processing in an fMRI study in pASD and highlighted the lower activation of right INS and higher activity in the AMY when compared with healthy controls. Additionally, an increased activation of LOC only in those parents with aloof personality was detected. Using fMRI, Greimel et al. [30] observed significant differences in the activation of AMY between pASD and controls during an empathy task, with parents displaying decreased activation. Notably, a positive correlation between INS activity and language score was also identified. Increased LOC and SMG activations that correlated with the level of language measures were also found during a MEG language auditory stimulation task [112] in pASD relative to controls. In a phonological processing fMRI task [29], greater hemodynamic response enhancement in several cortical regions, including insular cortex STG, SMG, as well as greater hemodynamic response suppression in the left lateralized postcentral gyrus, middle temporal gyrus (MTG), STG, and SMG was characteristic of pASD when compared to controls. Increased response in STG was also typical of pASD compared with healthy controls in the picture-naming MEG study by Buard et al. [113].

In our study, the higher autistic traits in fath-ASD are associated with lower connectivity in most of the regions that were identified in the abovementioned studies. Given that most of the functional studies have highlighted an increased activity of these regions during several social or auditory tasks, it is possible that the enhanced activation reflects a compensatory mechanism for the abnormal, reduced structural connectivity.

Most of the brain-behavior correlations that we observed were related to the “attention switching” domain of the AQ, suggesting that a wide range of brain areas contribute to modulating cognitive flexibility in fath-ASD. It should be noted that this item does include attention shifts, not only between non-social stimuli, but also between social stimuli. Thus, an impaired cognitive flexibility may reduce one’s ability to effectively attend to, process, and use social and emotional information [114]. Therefore, having a higher number of autistic traits may be related to displaying fewer social behaviors and experiencing more discomfort when doing so.

The correlations obtained for betweenness centrality confirm the relevance of the identified regions in the definition of the ASD phenotype. In particular, reduced centrality of left LOC, right AMY, and left SMG was related to increased autistic traits in the “imagination” domain, which is consistent with the role of LOC region in processing social stimuli [115], of AMY inferring mental states from faces [116], and of left SMG in linking symbols to their meaning [117]. Reduced centrality of bilateral INS was instead correlated with increased autistic traits in the “social skills” domain, which is in agreement with the reduced activation of the insula across several social cognitive task paradigms in individuals with ASD [111].

### 4.3. Overlap between Fathers and Their Children

Some of the regions that were identified as correlated with ADOS scores in the group of ASD probands overlap with those that were correlated to autistic traits in their fathers, thus suggesting an intergenerational transmission of neural substrates. In particular, the local efficiency of STG and cluster coefficient of FPO, ITG, and CNG were significantly negatively correlated with autistic features in ASD probands and positively correlated with autistic traits in fathers. This opposite direction of correlation is consistent with the inversion of connectivity pattern from childhood to adulthood that was previously discussed.

Previous MRI studies have suggested that abnormalities in the STG are highly implicated in ASD [118,119,120]. Interestingly, neuroimaging and neurophysiological studies show that, in the left hemisphere, the STG is implicated in language, while in the right hemisphere mediates spatial awareness and exploration [121]. In this study, we found a positive correlation between ADOS-CSS and right STG in children with ASD, which is in line with the impairment of language function in the clinical sample [122], and a negative correlation between the “attention switching” domain of the AQ and right STG in fath-ASD, revealing a deficit more related to the cognitive flexibility in fathers. These results are consistent with previous studies that identified the STG, the CNG, and the ITG as part of the circuit activated in response to switching the attention to an unattended stimulus [123].

The FPO plays a role in retrospective memory and in higher-order cognitive operations (e.g., decision making, planning, social/moral reasoning) [124], and structural abnormalities in this area has been previously linked to ASD [125]. Our results indicated a positive association with the social domain of ADOS in ASD probands and a negative correlation with the “attention switching” in fath-ASD, suggesting the involvement of this brain structure in mediating the ASD features in the two groups.

Moreover, the betweenness centrality of LOC, AMY, CNG, and MFG was correlated with autism severity in both ASD children and their fathers. The limbic system, including the cingulate gyrus, is related to emotion and social behaviors, and replicated evidences suggested that the disruption of this circuitry could be related to some of the behavioral deficits that were seen in individuals with ASD [126].

The different direction of correlations in ASD children and their fathers can be partially ascribed to their different ages, which implies a shift in the connectivity patterns, and partially to the different severity of ASD features (i.e., autistic disorder in children versus autistic traits in their fathers).

### 4.4. Strengths and Limitations

This is the first DWI study investigating the neurostructural correlates of BAP traits in fathers of individuals with ASD (fath-ASD). In addition, we enrolled children with ASD as well as their fathers, to allow for exploration of the intergenerational transmission of autistic features. Only one previous fMRI study [30] acquired both the ASD probands and their fathers, with the aim of exploring the intergenerational transmission of neural substrates. Overall, the results from these studies may help in elucidating the neural endophenotype of ASD and better clarifying the hereditary mechanisms that are involved in the various clinical dimension of ASD.

A further strength of this study is the use of the graph analysis approach to explore network characteristics and their behavioral correlates in children with ASD and in fath-ASD. This novel approach is providing interesting results for a comprehensive characterization of brain connectivity and it is improving our understanding of the brain organization in neurodevelopmental disorders as well as in other pathological conditions. Since ASD reports several white matter microstructure abnormalities, investigating the properties of the inter-regional correlations of white matter integrity may provide insight into the structural coherence of underlying white matter tracts in ASD and in fath-ASD. In particular, the characterization of the local properties of the structural connectivity can enhance our understanding of the correlations between white matter structure and behavioral impairments in the ASD endophenotype.

There are some limitations to our study that must be acknowledged. At first, the relatively low sample size only allows for partial conclusions regarding the common and distinct brain-behavior correlations in ASD preschoolers and in fath-ASD. With larger cohorts, it would be possible to better control for confounding factors, including IQ and psychiatric comorbidities, which could somehow affect the neuroanatomical underpinnings. However, the number of subjects that were included in this study is somewhat in line with previous MRI investigations on this topic [26,29,30,113]. In this context, our findings could add to the current literature by providing initial insight into DWI patterns in ASD individuals as well as in fath-ASD. Moreover, we did not include a control group, so the specificity of our results is unclear. Finally, as this was the first DTI study in fath-ASD, we did not have any a priori hypothesis allowing for restricting the number of comparisons between DTI measures for each brain region and different domains of autistic traits. As a result, FDR correction yielded a very conservative threshold with only two correlations surviving the correction. Since many of the extracted features or regions are likely to be highly dependent from each other, it is conceivable that an a priori limitation of the number of comparisons would have resulted in a higher number of significant correlations.

### 4.5. Future Directions

Future investigations should address the limitations of this and previous studies in pASD by including a larger sample of parents and a group of adults without a child with ASD, but comparable for autistic traits. In addition, multimodal imaging techniques that evaluate the structural and functional measures could help in elucidating the relationship between the neurostructural and neurofunctional correlates of autistic traits in ASD parents, including the potential compensatory neural activations to counter structural brain impairments. Moreover, studies assessing the BAP could also benefit from the assessment of multiple endophenotypes/biomarkers by collecting, in addition to neuroimaging data, immunological, biochemical, or neuropsychological information, thus addressing the cross talk among the different modalities [127]. Ultimately, the detection of common and distinct neuroimaging underpinnings in patients with ASD and in fath-ASD has the potential to bridge the gap between genes and clinical ASD features, and therefore to pave the way towards a better understanding of ASD etiopathogenesis.

## 5. Conclusions

Our results suggest that a significant association exists between BAP traits in fath-ASD and their white matter connectivity organization. Importantly, some aspects of the brain structure are shared by parents and their children with ASD, supporting their possible role as an endophenotype of the disorder. Conversely, several other patterns of brain connectivity are group-specific, with some regions being correlated with autistic features in children with ASD and others with BAP traits in fath-ASD. The specificity of these brain-behavior correlations could be due to the different age-range of the two groups of subjects, when considering that the connectome changes with age [86]. However, another possible, not mutually exclusive, explanation is that the regions exhibiting correlations with autistic traits in fath-ASD, but not in their children, have a more marginal role in defining the ASD endophenotype.

## Figures and Tables

**Table 1:** Demographic and clinical characteristics of the participants.

	**ASD Children (*n* = 16)**
Age (years)	3.1 (1.3)
ADOS, CSS	6.4 (1.3)
ADOS, SA	7.8 (1.4)
ADOS, RRB	6.1 (1.7)
Performance IQ ^a^	83.7 (13.7)
	**Fathers of children with ASD (*n* = 16)**
Age (years)	43.5 (5.1)
Autism-Spectrum Quotient (AQ), total	13.5 (7.2)
AQ range	4–28

Data are expressed as mean (SD). ADOS: Autism Diagnostic Observation Schedule; CSS: Calibrated Severity Score; SA: Social Affect; RRB: Restricted, Repetitive Behavior; AQ: Autism Quotient; ^a^ As assessed by the Griffiths Mental Developmental Scales (GMDS).

**Table 2:** Cortical and subcortical regions including in the final parcellation within each hemisphere.

Localization	Region	Abbreviation	Localization	Region	Abbreviation
Frontal lobe	Superior frontal gyrus	SFG		Transverse temporal cortex	TTC
	Middle frontal gyrus, rostral division	MFGrostr		Entorhinal cortex	ENC
	Middle frontal gyrus, caudal division	MFGcaud		Temporal pole	TPO
	Inferior frontal gyrus, pars opercularis	IFGoperc		Parahippocampal gyrus	PHG
	Inferior frontal gyrus, pars triangularis	IFGtriang	Occipital lobe	Lateral occipital cortex	LOC
	Inferior frontal gyrus, pars orbitalis	IFGorbit		Lingual gyrus	LING
	Orbitofrontal cortex, lateral division	ORBlat		Cuneus cortex	CUN
	Orbitofrontal cortex, medial division	ORBmed		Pericalcarine cortex	CAL
	Precentral gyrus	PCG	Cingulate Lobe	Cingulate cortex, rostral anterior division	CNGrostr
	Paracentral lobule	PCL		Cingulate cortex, caudal anterior division	CNGcaud
	Frontal pole	FPO		Cingulate cortex, posterior division	CNGpost
Parietal lobe	Superior parietal cortex	SPC		Cingulate cortex, isthmus division	CNGisthm
	Inferior parietal cortex	IPC	Insula	Insula	INS
	Supramarginal gyrus	SMG	Subcortical	Thalamus	THA
	Postcentral gyrus	POG		Caudate nucleus	CAU
	Precuneus cortex	PCN		Lenticular nucleus, putamen	PUT
Temporal lobe	Superior temporal gyrus	STG		Lenticular nucleus, pallidum	PAL
	Middle temporal gyrus	MTG		Hippocampus	HIP
	Inferior temporal gyrus	ITG		Amygdala	AMY
	Banks of the superior temporal sulcus	STS		Nucleus accumbens	ACC
	Fusiform gyrus	FFG		Cerebellar cortex	CRB

**Table 3:** Description of the global and local network measures.

Measure	Description
**Global measures**	
Global efficiency (EG)	Average of the inverse shortest path lengths, typically considered to be a measure of the network’s overall ability for information transfer and integrated processing.
Transitivity (T)	Ratio of triangles to triplets in the network. It is more robust compared to the average clustering coefficient.
Characteristic path length (CPL)	Average of the shortest path lengths between each pair of nodes in the network, where shortest path length is the minimum number of links that are passed through to get from one node to another node.
Small world propensity (SWP)	A measure of small-worldness for weighted graphs. Small-worldness is defined as a type of network that exhibits groups of highly clustered vertices (high clustering coefficient), with a limited number of edges connecting the vertex assemblies (low path length) [83].
**Nodal Measures**	
Local efficiency (EL)	Efficiency of a subgraph made up by the neighborhood of the node.
Clustering coefficient (CC)	Weighted sum of the number of links between the nearest neighbors of the node divided by the maximum possible amount of links between the nearest neighbors. It is a measure of the percentage of the node’s neighbors that are also connected to each other.
Betweenness centrality (BC)	Quantifies how many shortest paths between any two nodes pass through a given node. It is a measure for how important a given node is for the efficiency of the network.

**Table 4:** Significant correlations between nodal measures and psychological measures in children with Autism Spectrum Disorders (ASD), and in their fathers. Only correlations that survived false discovery rate (FDR) correction and/or were obtained in the same brain areas in the two groups are reported.

Children with ASD		Fathers of Children with ASD	
Local Efficiency (LE)			
**Brain region**	Significant interactions	Brain region	Significant interactions
Right PCL	ADOS-CSS: B = 0.78; F = 12.75, *p* = 0.006, η^2^ = 0.586 *	**Left CNG**isthm	Att. swi.: B = −1.09; F = 10.11, *p* = 0.01, η^2^ = 0.503
**Left CNGpost**	ADOS-CSS: B = 0.66; F = 8.05, *p* = 0.02, η^2^ = 0.472	**Right STG**	Att. swi.: B = −0.91; F = 5.32, *p* = 0.044, η^2^ = 0.384
**Right STG**	ADOS-CSS: B = 0.70; F = 11.75, *p* = 0.008, η^2^ = 0.566 * Age: B = −0.54; F = 6.79, *p* = 0.028, η^2^ = 0.430		
Left CAU	SA: B = 1.10; F = 19.66, *p* = 0.004, η^2^ = 0.766 *		
Cluster coefficient (CC)			
**Left CNG**post	ADOS-CSS: B = 0.76; F = 8.38, p = 0.018, η^2^ = 0.482	Right SFG	Att. swi.: B = −1.10; F = 12.61, *p* = 0.005, η^2^ = 0.558 *
Right **FPO**	SA: B = 0.92; F = 10.69, *p* = 0.017, η^2^ = 0.641	Right MFGrostr	Att. swi.: B = −1.03; F = 13.47, *p* = 0.004, η^2^ = 0.574 *
**Right ITG**	SA: B = 0.95; F = 14.21, *p* = 0.009, η^2^ = 0.703 GMDS: B = 0.59; F = 6.11, *p* = 0.048, η^2^ = 0.504	Left **FPO**	Att. swi.: B = −0.82; F = 5.67, *p* = 0.04, η^2^ = 0.362
Left CAU	SA: B = 1.11; F = 18.86, *p* = 0.005, η^2^ = 0.554 *	Left LOC	Att. swi.: B = −1.02; F = 10.92, *p* = 0.008, η^2^ = 0.522 *
		**Left CNG**isthm	Att. swi.: B = −1.15; F = 11.89, *p* = 0.006, η^2^ = 0.543 *
		**Right ITG**	Att. swi.: B = −0.71; F = 5.61, *p* = 0.039, η^2^ = 0.360
Betwenness centrality (BC)			
**Right MFGcaud**	SA: B = 0.97, F = 6.66; *p* = 0.04, η^2^ = 0.526	Left IFGoperc	Imm.: B = 1.00; F = 16.76, *p* = 0.002, η^2^ = 0.626 *
**Left CNG**post	SA: B = 0.91; F = 15.35, *p* = 0.008, η^2^ = 0.719 * RRB: B = −0.87; F = 15.06, *p* = 0.008, η^2^ = 0.683 *	Left POG	Age: B = 1.14; F = 14.46, *p* = 0.003, η^2^ = 0.591 *
		**Left LOC**	Imm.: B = −0.80; F = 6.41, *p* = 0.030, η^2^ = 0.391
Right PCG	ADOS-CSS: B = 0.91; F = 14.96, *p* = 0.008, η^2^ = 0.714 *	**Right MFGcaud**	Soc. skills: B = 0.89; F = 6.87, *p* = 0.025, η^2^ = 0.408
Left **AMY**	SA: B = −0.89; F = 10.41, *p* = 0.018, η^2^ = 0.662 Age: B = −0.75, *p* = 0.020, η^2^ = 0.609	Right MTG	Soc. skills: B = 1.00; F = 15.94, *p* = 0.003, η^2^ = 0.615 *
		Right MTG	Soc. skills: B = 1.00; F = 15.94, *p* = 0.003, η^2^ = 0.615 *
		**Left CNG**isthm	Imm.: B = −0.70; F = 5.41, *p* = 0.040, η^2^ = 0.351
		Right THA	Soc. skills: B = 0.98; F = 16.86, *p* = 0.002, η^2^ = 0.628 *
		Right **AMY**	Imm.: B = −0.76; F = 6.31, *p* = 0.030, η^2^ = 0.387

* Significant interaction after false discovery rate correction; In bold, regions for which correlations with clinical measures are shared by fathers and their children.

**Table 5:** Brain area for which significant correlations with clinical measures were found both in fathers and in their children.

Measure	Shared Brain Areas
Local efficiency (EL)	CNG, Right STG
Clustering coefficient (CC)	CNG, FPO, ITG
Betweenness centrality (BC)	CNG, LOC, MFG, AMY

## Supplementary Materials

The following are available online at https://www.mdpi.com/2077-0383/8/4/487/s1. S1. Nodal measures correlations in fathers and in their children; S2. Data analysis to understand if all the couples showed similar correlations; S3. Anatomical graphs for father and children; Table S1. Significant correlations between nodal measures extracted from the connectome weighted on the basis on the number of streamlines and psychological measures in children with ASD and in their fathers; Figure S1. Three dimensional sagittal and axial views of the anatomical graph in fathers (a) and in children (b) in which the size of the node represents the Local Efficiency (EL), while the thickness of the edges represents the strength of the connections (number of streamlines); Figure S2. Three dimensional sagittal and axial views of the anatomical graph in fathers (a) and in children (b) in which the size of the node represents the Cluster Coefficient (CC), while the thickness of the edges represents the strength of the connections (number of streamlines); Figure S3. Three dimensional sagittal and axial views of the anatomical graph in fathers (a) and in children (b) in which the size of the node represents the Betweennes Centrality (BC), while the thickness of the edges represents the strength of the connections (number of streamlines).
