# Supplementary material for: Brain Network Organization Correlates with Autistic Features in Preschoolers with Autism Spectrum Disorders and in Their Fathers: Preliminary Data from a DWI Analysis
Source: J Clin Med. 2019 Apr 10;8(4):487. doi: 10.3390/jcm8040487 (PMC6518033; doi:10.3390/jcm8040487)
Supplement: Supplementary file 1 [file jcm-08-00487-s001.pdf]

S1. Nodal measures correlations in fathers and in their children

Children

EL index. ADOS-CSS was significantly positively correlated with EL of bilateral PCL, left CNGpost and right STG. SA was also positively correlated with the left CAU, moreover it was also positively correlated with bilateral HIP and right IPC. RRB was significantly negatively correlated with left HIP and right IPC. A significant positive correlation was found between GMDS and the EL of the left CAU. Age also showed a significant negative correlation with EL of left CAU and of right STG.

CC index. For CC, significant correlations in most of the same areas were found. ADOS-CSS was positively correlated with CC of CNGpost. About ADOS subdomains, SA was positively correlated with several brain regions: left CAU, bilateral HIP, right FPO, right IPC and right ITG. RRB was correlated with CC of bilateral HIP. GMDS was positively correlated with left CAU, right ITG and right HIP.

BC index. ADOS-CSS was significantly positively correlated with the BC of the left IPC and right PCG, and negatively correlated with the BC of the left LOC. SA was significantly positively correlated with the BC of bilateral MFGcaud, left FFG, left LOC and the left CNGpost, and significantly negatively correlated with the BC of the left AMY and right PCG. RRB was negatively correlated with the BC of the left MFGcaud, left FFG, of the CNGpost, and positively correlated with left PCG. GMDS was negatively correlated with the BC of the left FFG and of left LOC.

Age showed a significant positive correlation with BC of the left FFG, left LOC, and a negative correlation with left AMY.

Table s1, left column, summarizes the significant correlations between ASD symptoms and brain network measures in ASD probands.

Fathers

EL index. No significant correlation between EL and total AQ were found. Conversely, the following significant correlations were observed.

The "attention switching" area of the AQ was negatively correlated with the EL of several brain regions: bilateral SFG, bilateral MFGrostr, left IFGoperc, left ORBlat, bilateral POG, left LOC, bilateral CNGisthm, right SMG, right PCN, right STG, right CUN and right INS. In addition, the "attention to details" of the AQ was significantly positive correlated with the EL of left IFGorbit while the "communication" area of AQ was positively correlated with right SFG.

CC index. Also for the CC the "attention switching" area of the AQ was negatively correlated with several brain regions: bilateral SFG, bilateral MFGrostr, left IFGoperc, left FPO FPO, left ORBlat, left POG, left LOC (, bilateral CNGisthm, right SMG, right PCN, right STG, right ITG, right CUN and right INS. Moreover the "communication" area of AQ was significantly positively correlated with the CC of right SFG and the "imagination" area of AQ was significantly negatively correlated with the CC of right ACC. Age significantly negatively correlated with the CC of right ACC.

BC index. Regarding the BC, a significant negative correlation was found between the "social skills" area of the AQ and the BC of both the left POG and bilateral INS, whereas a significant positive correlation between the "social skills" area of the AQ and the BC of the bilateral THA, right MFGcaud, and right MTG was detected. The correlation between "social skills" and BC of right thalamus survived the conservative FDR correction. The "attention switching" area of AQ was positively correlated with the BC of the left SPC. The "attention to details" area of AQ was positively correlated with the BC of the left SPC, right CNGisthm, and right PUT and negatively correlated with the BC of left POG. The "communication" area of AQ was negatively correlated with the BC of the left IFGoperc. The "imagination" area of AQ was also significantly, although positively, correlated with the BCN of the left IFGoperc: this correlation survived FDR correction. Moreover, "imagination" was positively correlated with the BC of the left SPC and negatively correlated with BC of left SMG, left LOC, bilateral CNGisthm, and right AMY. Age significantly correlated with the BC of left POG and with the left THA.

Table s1, right column, summarizes the significant correlations between BAP traits and brain network measures in fath-ASD.

Table 1. Significant correlations between nodal measures extracted from the connectome weighted on the basis on the number of streamlines and psychological measures in children with ASD and in their fathers.

**Children with ASD**		**Fathers of children with ASD**	
Local Efficiency (LE)			
**Brain region**	Significant interactions	Brain region	Significant interactions
Left PCL	ADOS-CSS: B=0.70; F=8.61, p=0.017, η^2=0.489	Left SFG	Att. swi.: B=-0.97; F=9.25, p=0.012, η^2=0.480
Right PCL	ADOS-CSS: B=0.78; F=12.75, p=0.006, η^2=0.586*	Right SFG	Att. swi.: B=-1.01; F=11.46, p=0.007, η^2=0.534*<br>Comm.: B=0.86; F=7.07, p=0.024, η^2=414
**Left CNGpost**	ADOS-CSS: B=0.66; F=8.05, p=0.02, η^2=0.472		
		Left IFGoperc	Att. swi.: B=-1.03; F=8.53, p=0.015, η^2=0.460
**Right STG**	ADOS-CSS: B=0.70; F=11.75, p=0.008, η^2=0.566*<br>Age: B=-0.54; F=6.79, p=0.028, η^2=0.430	Left IFGorbit	Att. det.: B=0.59; F=6.86, p=0.026, η^2=0.407
Left CAU	SA: B=1.10; F= 19.66, p=0.004, η^2=0.766*<br>GMDS: B=0.67; F=7.98, p=0.03, η^2=0.571<br>Age: B=0.59; F=7.22, p=0.036, η^2=0.546	Left ORBlat	Att. swi. B=-0.98; F=12.17, p=0.006, η^2=0.549
		Left LOC	Att. swi.: B=-0.91; F=6.89, p=0.025, η^2=0.408
Right IPC	SA: B=0.91; F=6.60, p=0.04, η^2=0.524,<br>RRB: B=-0.85, F= 6.24, p=0.045, η^2=0.510	Left MFGrostr	Att. swi.: B=-0.81; F=5.73, p=0.038, η^2=0.364
		Right MFGrostr	Att. swi.: B=-0.98; F=10.30, p=0.009, η^2=0.507
Left HIP	SA: B=0.92; F=6.93, p=0.039, η^2=0.536<br>RRB: B=-0.88; F= 6.84, p=0.04, η^2=0.533	Left POG	Att. swi.: B=-0.90; F=6.22, p=0.032, η^2=0.384
Right HIP	SA: B=0.92; F=7.45, p=0.034, η^2=0.554	Right POG	Att. swi.: B=-0.88; F=5.15, p=0.047, η^2=0.340
		**Left CNG**isthm	Att. swi.: B=-1.09; F=10.11, p=0.01, η^2=0.503
		Right CNGisthm	Att. swi.: B=-1.04; F=8.36, p=0.016, η^2=0.455
		Right SMG	Att. swi.: B=-0.89; F=6.27, p=0.031, η^2=0.385
		Right PCN	Att. swi.: B=-0.92; F=7.37, p=0.022, η^2=0.424
		**Right STG**	Att. swi.: B=-0.91; F=5.32, p=0.044, η^2=0.384
		Right CUN	Att. swi.: B=-0.80; F=5.78, p=0.037, η^2=0.384
		Right INS	Att. swi.: B=-0.91; F=7.08, p=0.024, η^2=0.415
Cluster coefficient (CC)			
**Left CNG**post	ADOS-CSS: B=0.76; F=8.38, p=0.018, η^2=0.482	Left SFG	Att. swi.: B=-1.00; F=8.67, p=0.015, η^2=0.464
Left CAU	SA: B=1.11; F=18.86, p=0.005, η^2=0.554*<br>GMDS: B=0.66, F= 7.32, p=0.035, η^2=0.759	Right SFG	Att. swi.: B=-1.10; F=12.61, p=0.005, η^2=0.558*<br>Comm.: B=0.87; F=6.84, p=0.024, η^2=0.406
Left HIP	SA: B=0.95; F=8.09, p=0.029, η^2=0.574<br>RRB: B=-0.91; F= 7.97, p=0.030, η^2=0.571	Left IFGoperc	Att. swi.: B=-1.06; F=9.33, p=0.01, η^2=0.498
		Left ORBlat	Att. swi.: B=-1.02; F=10.02, p=0.01, η^2=0.501
Right HIP	SA: B=0.96; F=10.77, p=0.017, η^2=0.643<br>RRB: B=-0.79; F=8.04, p=0.030, η^2=0.573<br>GMDS: B=0.88; F=10.17, p=0.019, η^2=0.629	Left MFGrostr	Att. swi.: B=-0.91; F=9.13, p=0.013, η^2=0.477
		Right MFGrostr	Att. swi.: B=-1.03; F=13.47, p=0.004, η^2=0.574*
Right IPC	SA: B=0.91; F= 6.23, p=0.047, η^2=0.509	Left **FPO**	Att. swi.: B=-0.82; F=5.67, p=0.04, η^2=0.362
Right **FPO**	SA: B=0.92; F=10.69, p=0.017, η^2=0.641	Left POG	Att. swi.: B=-0.88; F=5.10, p=0.049, η^2=0.334
**Right ITG**	SA: B=0.95; F=14.21, p=0.009, η^2=0.703<br>GMDS: B=0.59; F=6.11, p=0.048, η^2=0.504	Left LOC	Att. swi.: B=-1.02; F=10.92, p=0.008, η^2=0.522*
		**Left CNG**isthm	Att. swi.: B=-1.15; F=11.89, p=0.006, η^2=0.543*
		Right CNGisthm	Att. swi.: B=-1.04; F=8.36, p=0.015, η^2=0.455
		Right SMG	Att. swi.: B=-0.94; F=6.16, p=0.030, η^2=0.381
		Right PCN	Att. swi.: B=-1.00; F=8.91, p=0.014, η^2=0.471
		Right STG	Att. swi.: B=-1.00; F=6.66, p=0.027, η^2=0.400
		**Right ITG**	Att. swi.: B=-0.71; F=5.61, p=0.039, η^2=0.360

		Right CUN	Att. swi.: B=-0.81; F=6.77, p=0.026, η^2=0.404
		Right INS	Att. swi.: B=-0.92; F=6.05, p=0.034, η^2=0.377
		Right ACC	Imm.: B=-0.69; F=7.89, p=0.018, η^2=0.477<br>Age: B=-0.79; F=7.67, p=0.020, η^2=0.434
Betwenness centrality (BC)			
Left IPC	ADOS-CSS: B=0.85; F=8.61; p=0.026, η^2=0.589	Left IFGoperc	Imm.: B=1.00; F=16.76, p=0.002, η^2=0.626*<br>Comm: B=-0.97; F=10.02, p=0.010, η^2=0.500
**Left LOC**	ADOS-CSS: B=-.60; F=9.93, p=0.020, η^2=0.623<br>SA: B=0.72; F=11.37, p=0.015, η^2=0.655<br>GMDS: B=-0.64, p=0.019, η^2=0.630<br>Age: B=0.63; F=10.21, p=0.016, η^2=0.649	Left POG	Soc. skills: B=-0.67; F=6.15, p=0.033, η^2=0.381<br>Att. det.: B=0.49; F=5.47, p=0.040, η^2=0.353<br>Age: B=1.14; F=14.46, p=0.003, η^2=0.591*
Left MFGcaud	SA: B=0.97; F=9.83, p=0.020, η^2=0.621<br>RRB: B=-0.98; F=12.93, p=0.011, η^2=0.503	Left SPC	Att. swi.: B=0.83; F=6.79, p=0.026, η^2=0.404<br>Att. det.: B=-0.51; F=5.07, p=0.048, η^2=0.336<br>Imm.: B=0.64; F=5.50, p=0.040, η^2=0.355
**Right MFGcaud**	SA: B=0.97, F=6.66; p=0.04, η^2=0.526	Left SMG	Imm.: B=-0.84; F=8.77, p=0.014, η^2=0.467
Left FFG	SA: B=0.61; F=9.22, p=0.023, η^2=0.606<br>RRB: B=-0.48; F=6.07, p=0.049, η^2=0.503<br>GMDS: B=-0.59; F=9.68, p=0.021, η^2=0.617<br>Age: B=0.48, p=0.036, η^2=0.548	**Left LOC**	Imm.: B=-0.80; F=6.41, p=0.030, η^2=0.391
		**Right MFGcaud**	Soc. skills: B=0.89; F=6.87, p=0.025, η^2=0.408
**Left CNG**post	SA: B=0.91; F=15.35, p=0.008, η^2=0.719*<br>RRB: B=-0.87; F=15.06, p=0.008, η^2=0.683*	Right MTG	Soc. skills: B=1.00; F=15.94, p=0.003, η^2=0.615*
		**Left CNG**isthm	Imm.: B=-0.70; F=5.41, p=0.040, η^2=0.351
Left PCG	RRB: B=0.91; F=6.94, p=0.039, η^2=0.536	Right CNGisthm	Att. det.: B=0.63; F=7.90, p=0.018, η^2=0.441<br>Imm.: B=-0.62; F=5.38, p=0.040, η^2=0.350
Right PCG	ADOS-CSS: B=0.91; F=14.96, p=0.008, η^2=0.714<br>SA: B=-0.90; F=6.66, p=0.014, η^2=0.526	Right PCN	Imm.: B=-0.81; F=5.38, p=0.040, η^2=0.350
Left **AMY**	SA: B=-0.89; F=10.41, p=0.018, η^2=0.662<br>Age: B=-0.75, p=0.020, η^2=0.609	Left INS	Soc. skills: B=-0.81; F=6.45, p=0.029, η^2=0.392
		Right INS	Soc. skills: B=-0.78; F=5.43, p=0.040, η^2=0.352
		Left THA	Soc. skills: B=0.99; F=10.17, p=0.010, η^2=0.504<br>Age: B=-0.87; F=6.39, p=0.030, η^2=0.390
		Right THA	Soc. skills: B=0.98; F=16.86, p=0.002, η^2=0.628*
		Right PUT	Att. det.: B=-0.73; F=10.11, p=0.010, η^2=0.503
		Right **AMY**	Imm.: B=-0.76; F=6.31, p=0.030, η^2=0.387

* Significant interaction after false discovery rate correction.

S2. Data analysis to understand if all the couples showed similar correlations

To understand if all the couples exhibit the same degree of correlation between DTI and clinical measures we performed the following analysis. For the GLM analysis in which significant DTI-clinical association were found we saved residuals, which express the distance between each data point and the regression line, and so it is an indication of the "degree" of correlation. Then we calculate the Pearson's correlation coefficient between each couple (father-children) of residuals obtained for the same brain regions. For example, we calculated the correlation between residual of the GLM analysis for EL left CNG isthm in fathers and EL left CNG post in children. We expected that if all the couples showed a similar degree of DTI-clinical correlation, the correlation between residuals would be significant.

We obtained the following results:

EL Left CNG (f:isthm, c:post) r=0.1, p=0.80
EL right STG r=0.72, p=0.008*

CC FPO (f:left, c:right) r=0.03, p=0.91
CC right ITG r=0.52, p=0.04*
CC CNG (f:isthm, c:post) r=0.26, p=0.33

BC Left LOC r=0.03, p=0.91
BC right MFGcaud r=0.19, p=0.47
BC CNG (f: right isthm, c:left post) r=0.51, p=0.04*
BC AMY (f:right, c:left) r=0.15, p=0.56

Thus, only for EL Left CNG, CC right ITG and BC CNG we obtained significant correlations meaning that for these measures the couples show a similar degree of DTI-clinical correlation while for the other measures there could be a difference in the extent to which DTI correlates with clinical measures among the different couples. In a future study, with a larger sample, we could better explore the reasons of these differences.

S3. Anatomical graphs for father and children

To better visualize the anatomical regions that were significantly associated with autistic traits in fathers and their children, the results are plotted in Figures s1-s3 by superimposing the connectivity graphs onto the brain and rendering them in different views. These figures were visualized with the BrainNet Viewer ([1], https://www.nitrc.org/projects/bnv/). Each line in Figures s1-s3 show a connection weighted by the number of streamlines. Each node is placed in the brain according to its anatomical location, and the size of the node reflects the values of the different measures extracted (EL, CC, and BC). The brain areas that are shared by fathers and children are represented in a different color in respect to the other ones.

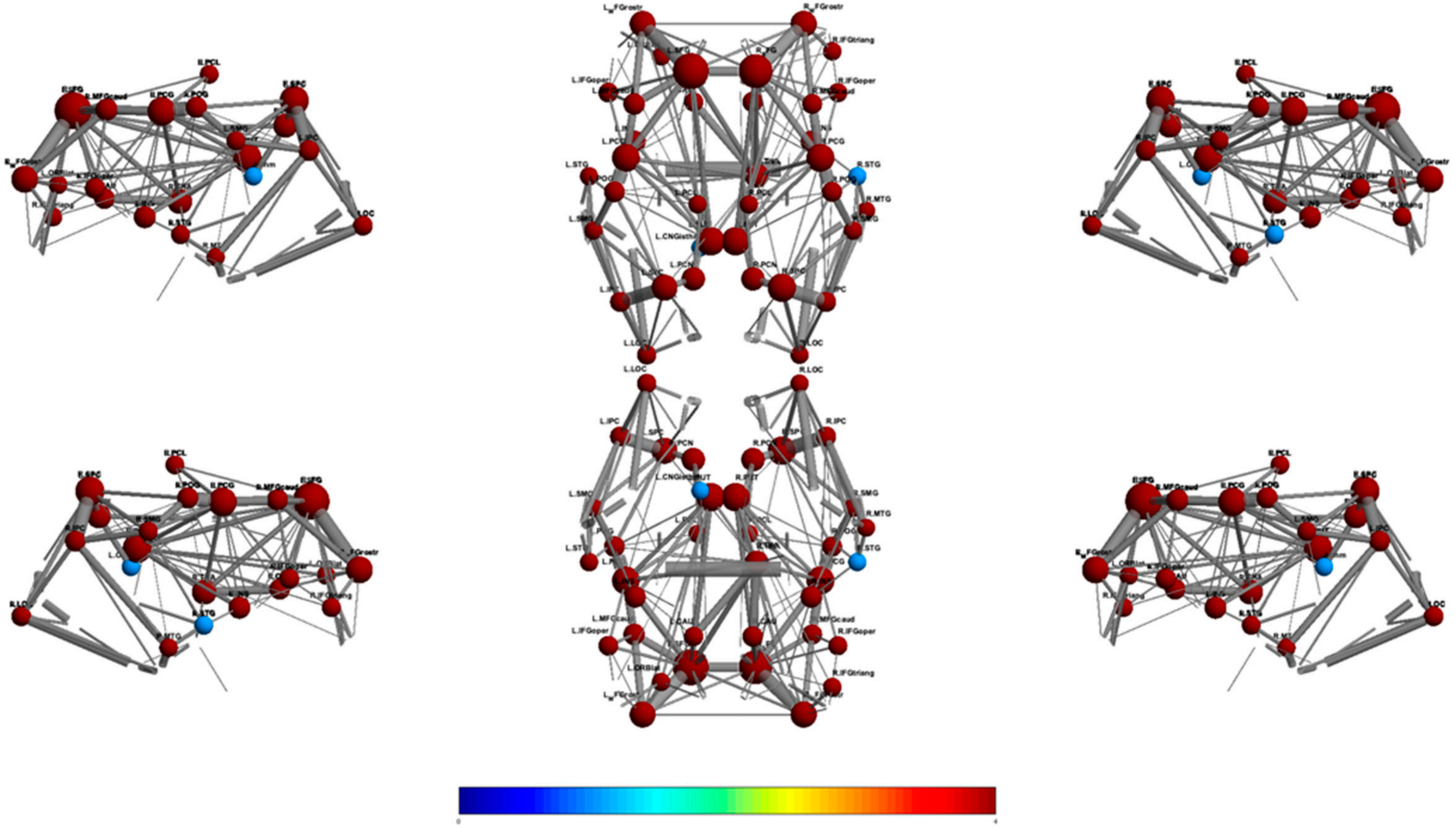

a)

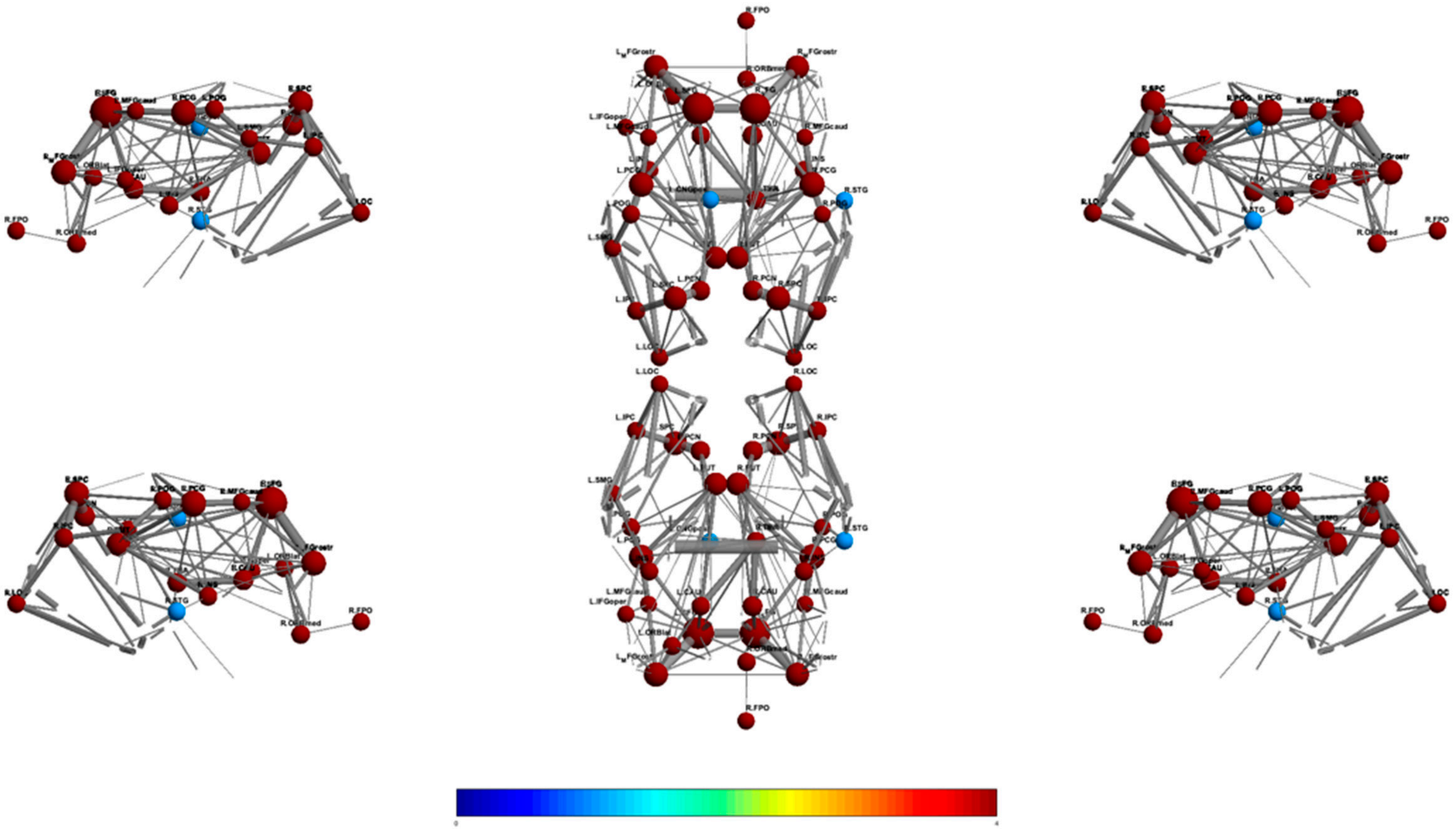

b)

Figure 1. Three dimensional sagittal and axial views of the anatomical graph in fathers (**a**) and in children (**b**) in which the size of the node represents the Local Efficiency (EL), while the thickness of the edges represents the strength of the connections (number of streamlines). For visualization purpose, only the nodes with an EL value above the threshold of 1.40 and the edges with strength above the threshold of 3500 are represented. In cyan, the nodes for which correlations with clinical measures are shared by fathers and their children.

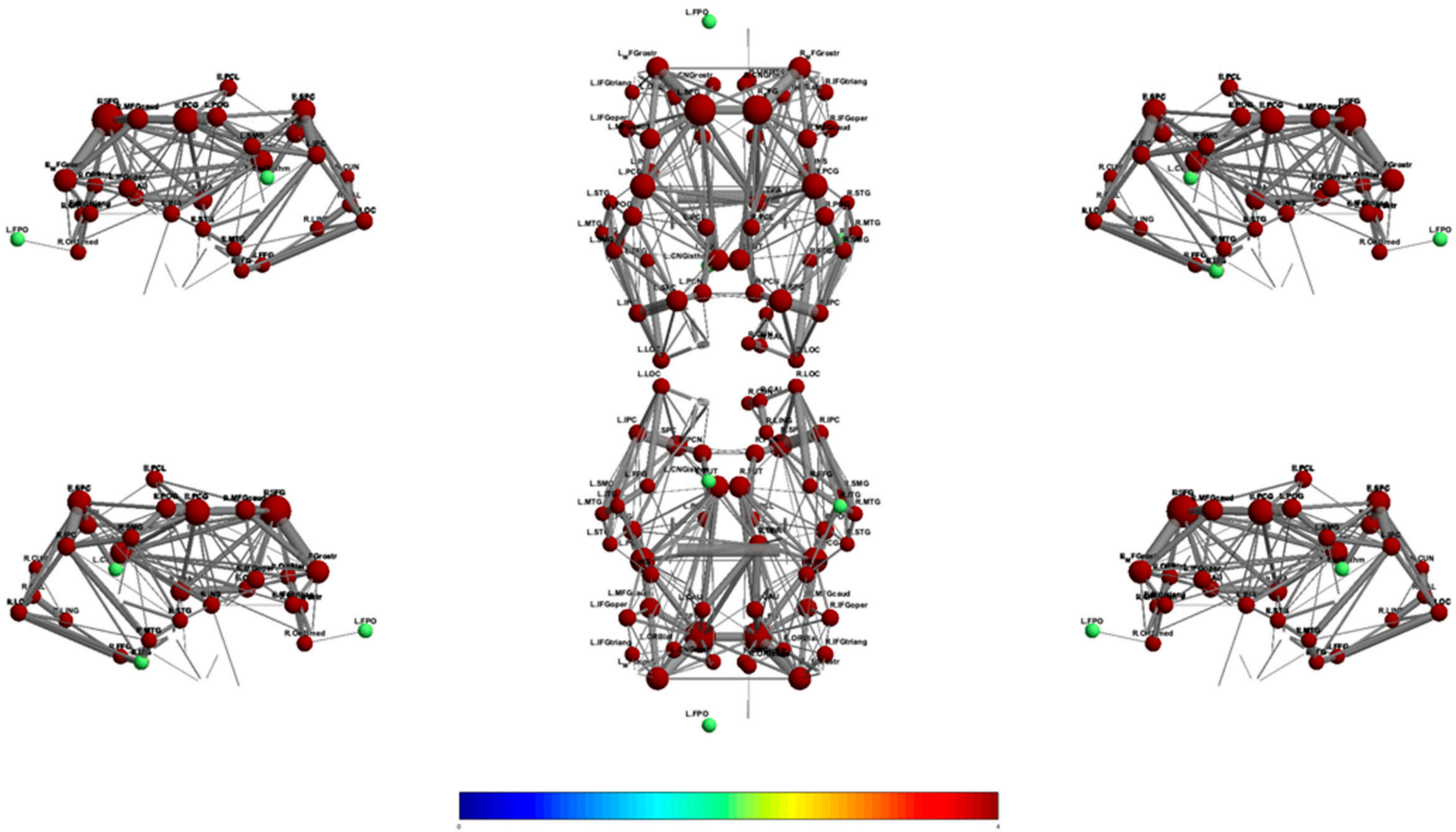

a)

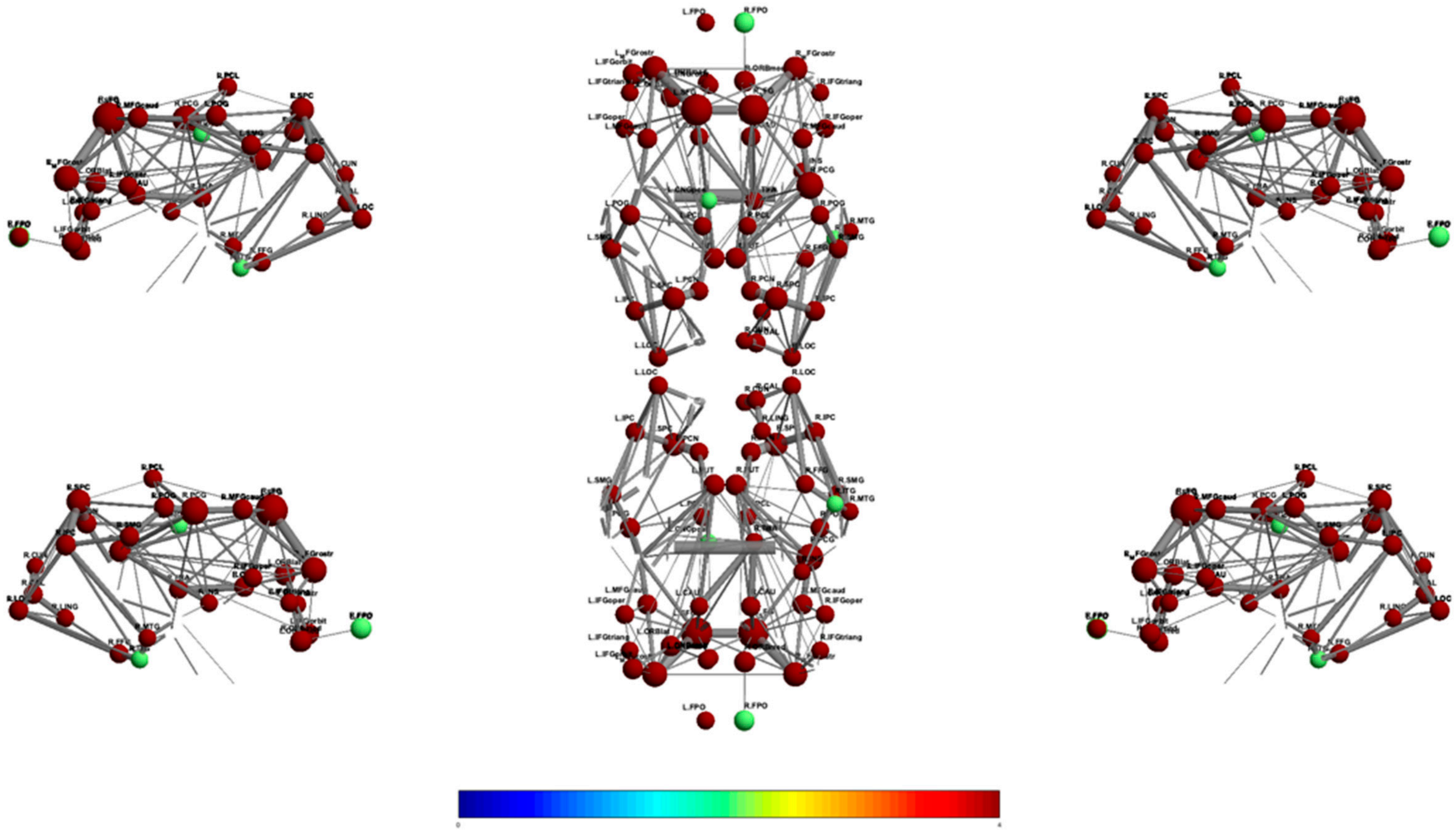

b)

Figure 2. Three dimensional sagittal and axial views of the anatomical graph in fathers (a) and in children (b) in which the size of the node represents the Cluster Coefficient (CC), while the thickness of the edges represents the strength of the connections (number of streamlines). For visualization purpose, only the nodes with a CC value above the threshold of 0.65 and the edges with strength above the threshold of 3500 are represented. In green, the nodes for which correlations with clinical measures are shared by fathers and their children.

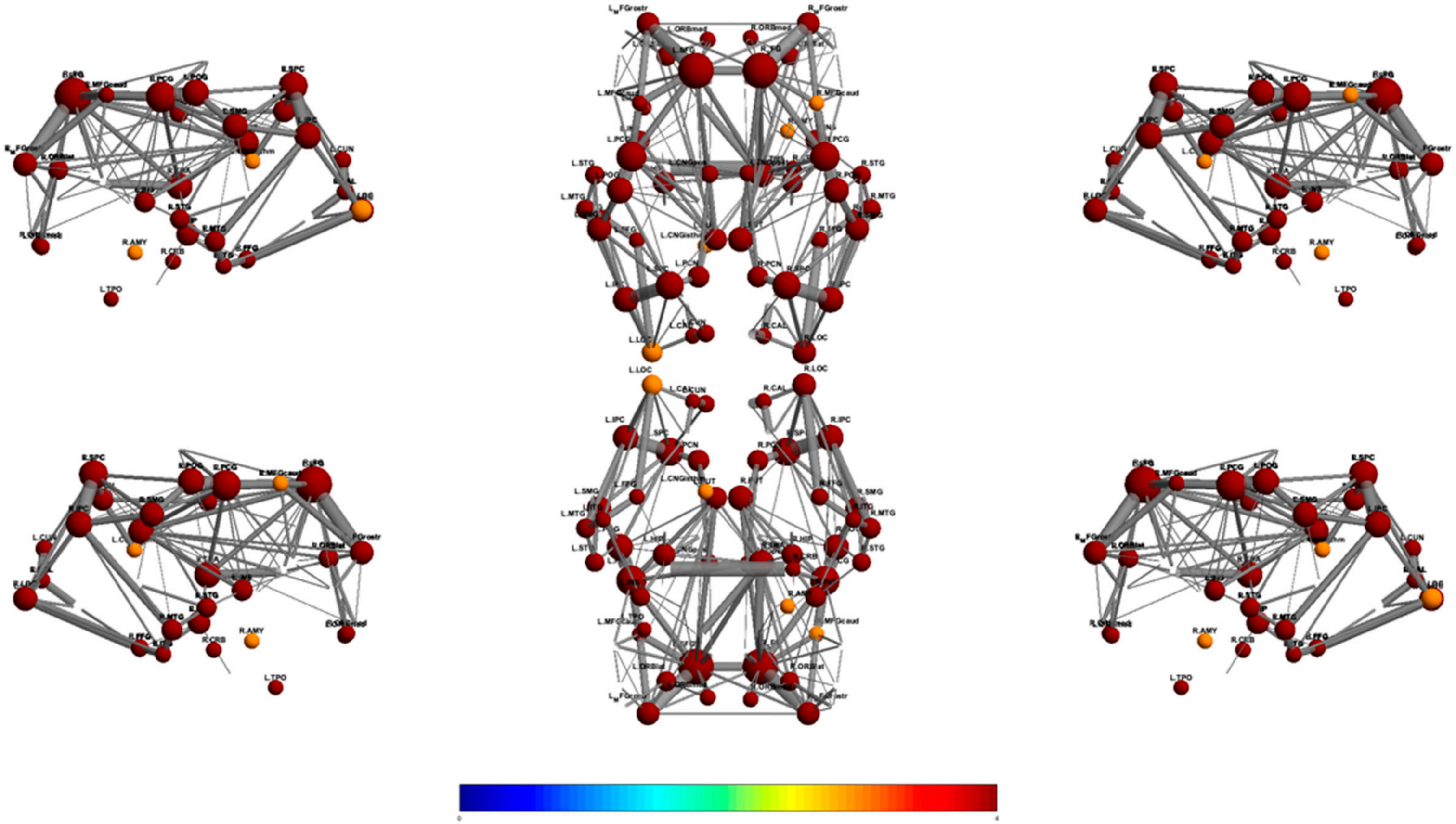

a)

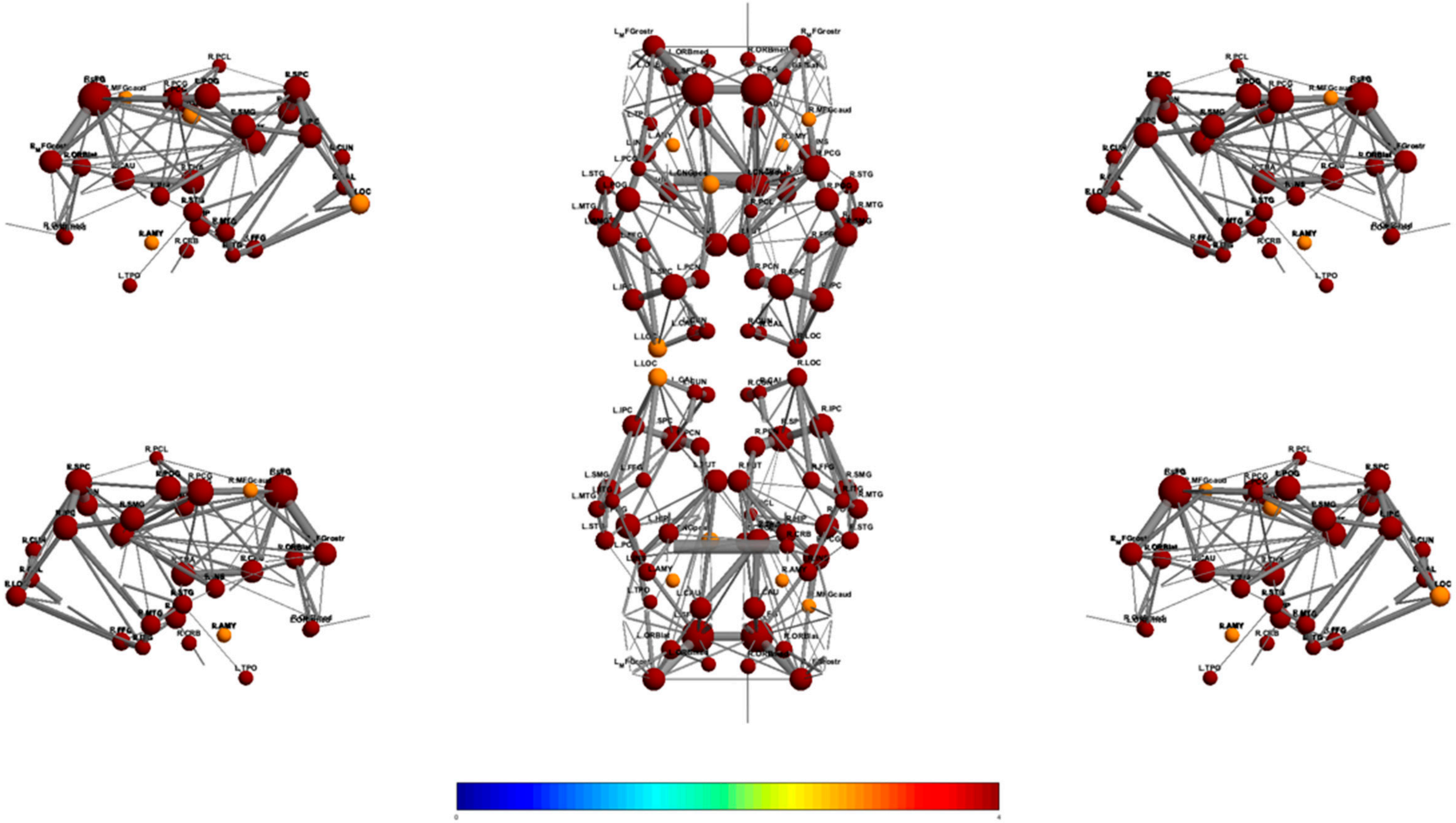

b)

Figure 8. Three dimensional sagittal and axial views of the anatomical graph in fathers (a) and in children (b) in which the size of the node represents the Betweennes Centrality (BC), while the thickness of the edges represents the strength of the connections (number of streamlines). For visualization purpose, only the nodes with a BC value above the threshold of 1.0 and the edges with strength above the threshold of 3500 are represented. In orange, the nodes for which correlations with clinical measures are shared by fathers and children.

Reference

1. Xia, M.; Wang, J.; He, Y. BrainNet Viewer: A Network Visualization Tool for Human Brain Connectomics. *PLoS ONE* **2013**, *8*, e68910.
